# Youth and Young Adult National Overdose Rates: A Descriptive, Ecological Cross-Sectional Time-Trend Analysis of Drug Overdose Mortality in 10-24-Year-Olds in the United States

**DOI:** 10.7759/cureus.109677

**Published:** 2026-05-26

**Authors:** Jane Wakefield, Chris Sun, Sina Ramtin, Anthony Castro, Asif M Ilyas

**Affiliations:** 1 Opioid Research and Education, Drexel University College of Medicine, Philadelphia, USA; 2 Opioid Research and Education, Rothman Institute Foundation for Opioid Education and Research, Philadelphia, USA; 3 Opioid Research and Education, Rothman Institute Foundation for Opioid Research and Education, Philadelphia, USA; 4 Opioid Research and Education, Rothman Institute Foundation for Opioid Research and Education, Philadephia, USA

**Keywords:** center for disease control, covid-19, drug overdose, overdose prevention, pediatric overdose, public health research

## Abstract

Introduction

Drug overdose (OD) mortality continues to be a public health crisis in the United States (US). The ongoing fourth wave of the opioid crisis necessitates continued monitoring of contemporary drug OD trends, particularly among vulnerable populations, such as the youth and young adults (YYAs) aged 10-24 years old. Furthermore, the COVID-19 pandemic may have exacerbated the opioid crisis in this specific population, warranting further investigation. Thus, understanding the current dynamics of drug OD among YYAs can offer descriptive data to inform targeted intervention and resource allocation. As such, this ecological cross-sectional time-trend analysis aims to evaluate OD trends among those aged 10-24 years in the US from 2018 to 2023, with additional analyses focusing on region, race and ethnicity, and sex-specific trends. This study hypothesized that while OD death rates may have declined since the peak of the COVID-19 pandemic, current rates are still higher than pre-pandemic rates.

Methods

A primary query using the United States Centers for Disease Control and Prevention’s (CDC) Wide-ranging ONline Data for Epidemiologic Research (WONDER) database yielded underlying cause of death data for those aged 10 to 24 years due to unintentional drug OD between 2018 and 2023. OD death rates per 100,000 were then analyzed by US geographical divisions and regions, race, ethnicity, and sex. For analysis, non-inferential, descriptive statistics were used. Crude OD rates were presented with 95% confidence intervals.

Results

Unintentional OD deaths peaked during the COVID-19 pandemic, and across the majority of the country, rates continue to be higher post-pandemic than pre-pandemic. Although the YYA OD death rate rose sharply between 2019 and 2020 from 6.6 [6.4-6.8] to 10.3 [10.1-10.6] per 100,000 individuals, the rate peaked at 10.5 [10.3-10.8] per 100,000 in 2021. While declining since 2021, the OD mortality rate remained higher in 2023 (8.3 [8.1-8.5]) than in 2018 (6.4 [6.2-6.6]) and 2019 (6.6 [6.4-6.8]). YYA males overall had higher rates of OD, yet the percent changes of OD death rates were higher among YYA females across most census divisions from the pre-pandemic period to the peri/post-pandemic period. Furthermore, American Indian and Alaska Native (AI/AN) YYAs were particularly impacted, with males and females experiencing an increase in OD death rate from 8.7 [7.0-10.7] pre-pandemic to 14.5 [12.8-16.1] peri/post-pandemic and 3.5 [2.4-4.9] pre-pandemic to 9.1 [7.7-10.4] peri/post-pandemic, respectively. Black or African American YYAs similarly exhibited an over twofold increase in crude OD death rate, from 5.5 [5.0-5.9] pre-pandemic to 13.2 [12.6-13.7] peri/post-pandemic for males, and from 2.5 [2.2-2.8] pre-pandemic to 6.2 [5.9-6.6] peri/post-pandemic for females.

Conclusion

Youth and young adults continue to be impacted by the ongoing opioid crisis and drug OD, highlighting an ongoing public health concern that must be addressed. Rates of unintentional OD deaths remain elevated above pre-pandemic levels despite decreasing since the peak of the pandemic. Addressing current OD trends among YYAs populations can maximize population-specific resource allocation, in hopes of reducing preventable, unintentional deaths among this vulnerable population.

## Introduction

The overdose (OD) and opioid crisis continue to be a major public health issue in the United States (US). Prescription opioids continue to offer a starting point for opioid use disorder in all age groups, with the U.S consuming 80% of the global opioid supply [1]. Illicitly manufactured synthetic fentanyl is also a growing concern, contributing to unintentional OD death [2,3]. Opioids have become the most common cause of fatal poisonings within the last decade, with synthetic opioids and illicitly manufactured fentanyl contributing to a significant proportion of unintentional ODs among children and youth [2-6]. In recent years, the epidemic has entered a “fourth wave,” characterized by increasing deaths involving illicit fentanyl in combination with other adulterants, such as xylazine and medetomidine [7]. Given the polysubstance landscape, continued investigation into OD trends is imperative.

More broadly and beyond opioids, drug OD is now one of the leading causes of death among children and adolescents [2,4,8]. In younger children, where unintentional poisoning rates are significant [9], this could be due to the improper storage of medications in a household [10-12]. Among youth and young adults (YYAs), drug use and overdose risk may be compounded by developmental and behavioral factors [13,14]. Based on 2024 data from the 2025 Monitoring the Future annual report, the lifetime prevalence of illicit drug use among 8th, 10th, and 12th graders was 15.1%, 23.7%, and 36.8%, respectively [15]. 

More recently, the COVID-19 pandemic has caused disruptions to everyday life, including heightened social isolation, increased financial and resource insecurity, intensified mental health concerns, and impacted healthcare access [16-18]. Drug markets also changed due to the COVID-19 pandemic, with increased contamination of adulterants within drug supplies [19]. Such dynamics may also have affected contemporary OD patterns, warranting close evaluation.

Despite the scope of the OD crisis in the U.S., YYA fatal OD data remains limited compared to older adult OD data. This is particularly concerning, since it is known that a significant percentage of adult drug use begins in adolescent years, and the onset of drug use during an individual’s younger years can impact adulthood [20, 21]. Analyzing epidemiologic data is important for developing effective, data-driven policies and resources to curb preventable deaths among YYAs. Such data is also relevant to clinicians and healthcare systems, including nursing and emergency care teams, who are often involved in acute OD management and prevention efforts.

Therefore, the purpose of this ecological cross-sectional time-trend study was to evaluate national trends in unintentional OD death rates among YYAs aged 10-24 years in the US from 2018 to 2023, and to compare the pre-pandemic (2018-2019) and peri/post-pandemic (2020-2023) periods. In doing so, the study aims to assess drug ODs occurring in YYAs over time across the broad spectrum of exposures affecting this population, including accidental pediatric ingestion to substance use-related OD. The second aim of this study is to evaluate OD deaths across US geographical regions, sex, race, and ethnicity to identify the populations most impacted. The study hypothesis was that while rates of unintentional YYA OD deaths have decreased since the height of the COVID-19 pandemic in 2020-2021, rates are still higher than before the pandemic. These findings may help guide clinicians, public health officials, and policymakers in developing targeted interventions to reduce preventable OD deaths amid the evolving crisis.

## Materials and methods

Data source 

For this ecological cross-sectional time-trend analysis, data were compiled from the United States Centers for Disease Control and Prevention (CDC) Wide-ranging ONline Data for Epidemiological Research (WONDER) database, an ad hoc query system for public health data (CDC, Atlanta, USA). The primary query utilized the dataset labeled "2018-2023: Underlying Cause of Death by Single-Race Categories." Data use restrictions were accepted and followed. Due to the public and de-identified nature of this data, Institutional Review Board review and approval were determined not to be required and therefore exempt. 

Data stratification

Regions and corresponding states were defined as the following: New England (Connecticut, Massachusetts, Maine, New Hampshire, Rhode Island, Vermont), Middle Atlantic (New Jersey, New York, Pennsylvania), East North Central (Illinois, Indiana, Michigan, Ohio, Wisconsin), West North Central (Iowa, Kansas, Minnesota, Missouri, Nebraska, North Dakota, South Dakota), South Atlantic (Delaware, District of Columbia, Florida, Georgia, Maryland, North Carolina, South Carolina, Virginia, West Virginia), East South Central (Alabama, Kentucky, Mississippi, Tennessee), West South Central (Arkansas, Louisiana, Oklahoma, Texas), Mountain (Arizona, Colorado, Idaho, Montana, Nevada, New Mexico, Utah, Wyoming), and Pacific (Alaska, California, Hawaii, Oregon, Washington).

Further extrapolation included crude OD death rates across different races, ethnicities, and sexes, all defined by the CDC. Race and ethnicity were defined by U.S. Census categories: American Indian or Alaska Native (AI/AN), Native Hawaiian or Other Pacific Islander (NH/OPI), Asian, Black or African American, White, more than one race, and Non-Hispanic or Hispanic. Sex was divided into male or female. Due to data suppression constraints and to protect privacy, analyses of crude death rates between different racial groups excluded decedents identified as “Native Hawaiian or Other Pacific Islander” and “More than one race.”

Death rate data were also evaluated between two defined time periods, with years 2018-2019 defined as the time before the COVID-19 pandemic (pre-pandemic) and years 2020-2023 defined as the period during and after the peak of the COVID-19 pandemic (peri/post-pandemic). Although pre-pandemic and peri/post-pandemic groupings were used for analyses involving US census divisions, regions, race, and ethnicity, the annual OD death rates were also presented to capture potential year-by-year fluctuations better.

Reported data

All deaths included were classified as unintentional overdose (ICD-10 codes X40-X44). Crude death rate data were reported from each year from 2018 to 2023. Deaths among those aged 10-14, 15-19, and 20-24 years were combined to define the YYA study population. Despite developmental and risk-behavior differences among children aged 10-14 years, adolescents aged 15-19 years, and young adults aged 20-24 years, these 5-year age groups were aggregated to provide a robust denominator. Analyses based on US census regions and divisions stratified by 5-year age groups were not feasible due to data-suppression constraints.

The CDC WONDER database automatically suppresses data points that may pose a privacy threat, including instances with fewer than 10 deaths. Where the data were flagged as “suppressed,” the values were not reported to protect the decedent's privacy. However, these counts were included in the higher-level national aggregates provided by the CDC, where data suppression was minimized.

Analysis 

Crude death rates were calculated per 100,000 individuals using the population estimates returned by the CDC WONDER queries for the corresponding years. All crude rates were presented with 95% confidence intervals. Percent change in death rates between the pre-pandemic and peri/post-pandemic periods was calculated by dividing the difference in death rates by the pre-pandemic rate, then multiplying by 100. Given the nature of this study, no formal statistical tests were performed. Instead, non-inferential, descriptive statistics were utilized. 

## Results

**Figure 1:**
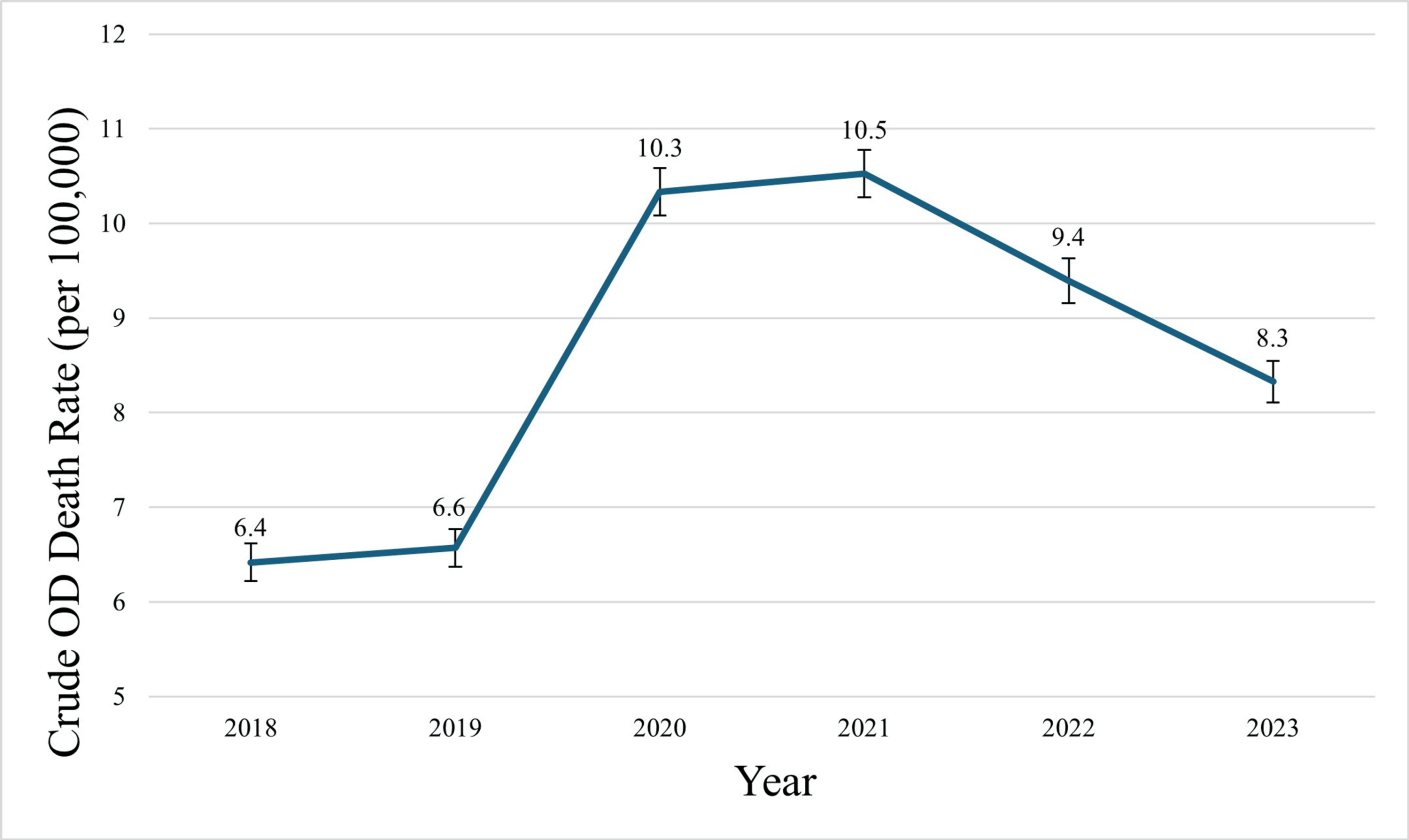
Crude overdose (OD) death rates from 2018 to 2023 among youth and young adults aged 10 to 24 years. Crude death rates are presented per 100,000 individuals with 95% confidence intervals.

Across the US, the overall YYA OD mortality rate rose sharply between 2019 and 2020. This number continued to rise, reaching its peak of 10.5 OD mortalities per 100,000 individuals in 2021. While declining since 2021, the OD mortality rate remained higher in 2023 (8.3) than in 2018 (6.4) and 2019 (6.6) (Table 1, Figure 1).

**Table 1:** Crude overdose death rates from 2018-2023 for youth and young adults aged 10-24 years old. Crude death rates are presented per 100,000 individuals with 95% confidence intervals. Absolute counts are provided to aid in the interpretation of rate stability and variability.

Year	Deaths	Population	Crude Rate [95% CI]
2018	4098	63850327	6.4 [6.2-6.6]
2019	4171	63485778	6.6 [6.4-6.8]
2020	6543	63310107	10.3 [10.1-10.6]
2021	6792	64536447	10.5 [10.3-10.8]
2022	6127	65231410	9.4 [9.2-9.6]
2023	5389	64721216	8.3 [8.1-8.5]

**Figure 2:**
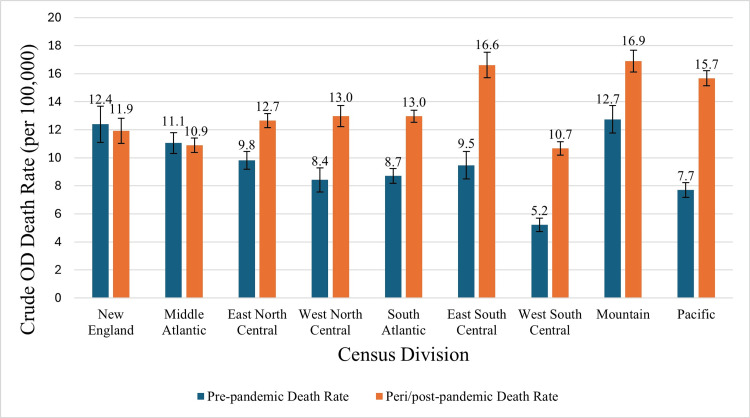
Pre-pandemic (2018-2019) and peri/post-pandemic (2020-2023) overdose (OD) death rates among male youth and young adults aged 10 to 24 years by census division. Crude death rates are based on population counts of >1,000,000 individuals and presented per 100,000 individuals with 95% confidence intervals.

**Figure 3:**
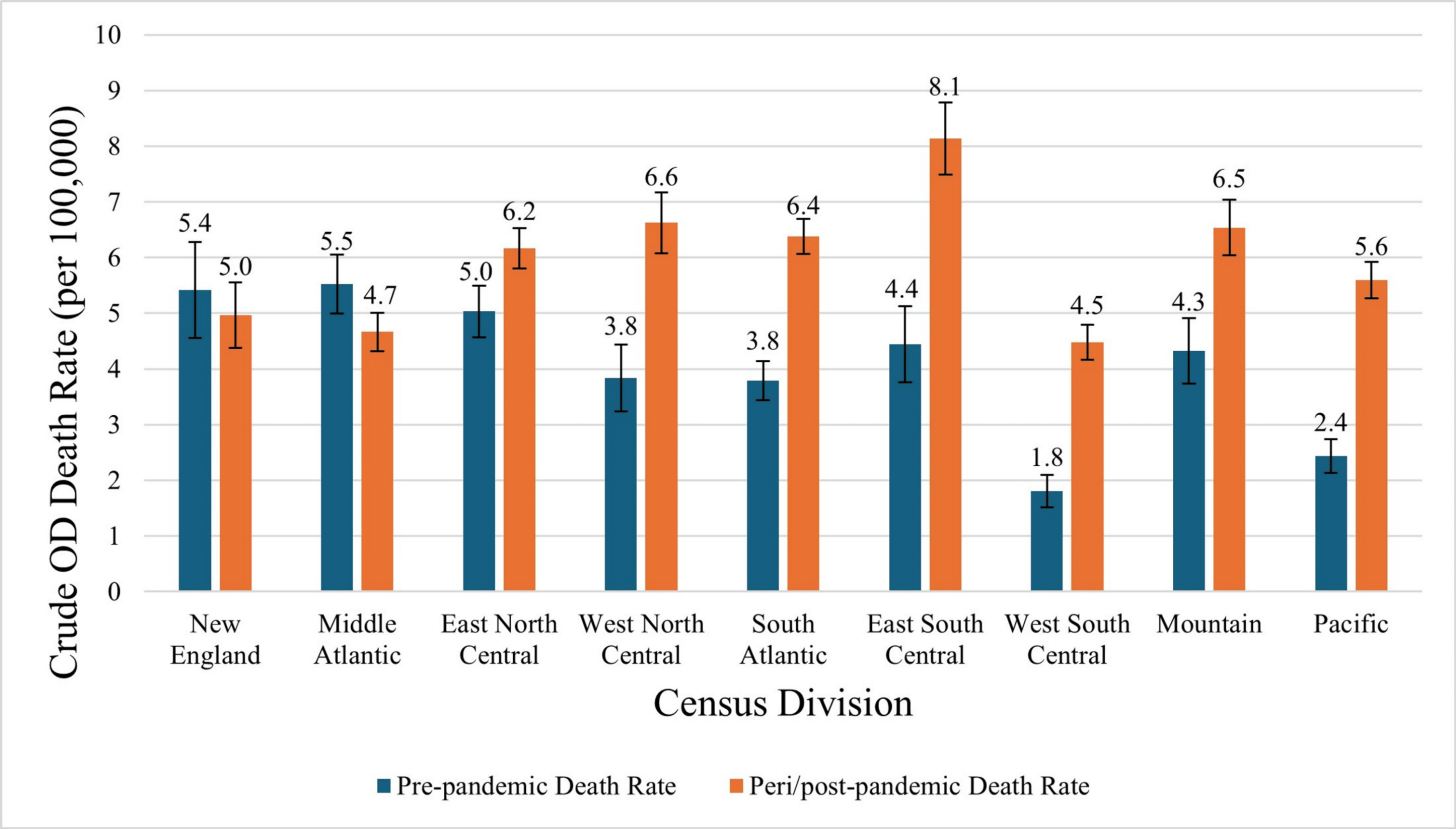
Pre-pandemic (2018-2019) and peri/post-pandemic (2020-2023) overdose (OD) death rates among youth and young adult females aged 10 to 24 years by census division. Crude death rates are based on population counts of >1,000,000 individuals and presented per 100,000 individuals with 95% confidence intervals.

Analysis was further divided by geography, sex, and racial/ethnic categories. With the exception of the New England and Middle Atlantic divisions, US census divisions experienced increases in crude death rates for both males and females during this period (Table 2, Figure 2, Table 3, Figure 3). The West South Central region demonstrated the most dramatic increase, with male YYA OD death rates rising by 104.3% and female YYA OD death rates rising by 148.1%. 

**Table 2:** Percent change of crude overdose death rates among male youth and young adults aged 10 to 24 years in the US by census division, pre-pandemic (2018-2019) to peri/post-pandemic (2020-2023). Crude death rates are based on base population counts of at least 1,000,000 individuals and presented per 100,000 individuals with 95% confidence intervals.

Census Division	Pre-Pandemic Rate [95% CI]	Peri/Post-Pandemic Rate [95% CI]	Percent Change
New England	12.4 [11.1-13.7]	11.9 [11.0-12.8]	-3.7
Middle Atlantic	11.1 [10.3-11.8]	10.9 [10.4-11.4]	-1.5
East North Central	9.8 [9.2-10.4]	12.7 [12.1-13.2]	29.0
West North Central	8.4 [7.6-9.3]	13.0 [12.2-13.7]	54.0
South Atlantic	8.7 [8.2-9.2]	13.0 [12.5-13.4]	48.9
East South Central	9.5 [8.5-10.4]	16.6 [15.7-17.5]	75.6
West South Central	5.2 [4.7-5.7]	10.7 [10.2-11.1]	104.3
Mountain	12.7 [11.8-13.7]	16.9 [16.1-17.7]	32.6
Pacific	7.7 [7.2-8.2]	15.7 [15.1-16.2]	103.3

**Table 3:** Percent change of crude overdose death rates among female youth and young adults aged 10 to 24 years in the US by census division, pre-pandemic (2018-2019) to peri/post-pandemic (2020-2023). Crude death rates are based on base population counts of at least 1,000,000 individuals and presented per 100,000 individuals with 95% confidence intervals.

Census Division	Pre-pandemic Rate [95% CI]	Peri/Post-Pandemic Rate [95% CI]	Percent Change
New England	5.4 [4.5-6.3]	5.0 [4.4-5.5]	-8.3
Middle Atlantic	5.5 [5.0-6.1]	4.7 [4.3-5.0]	-15.6
East North Central	5.0 [4.6-5.5]	6.2 [5.8-6.5]	22.6
West North Central	3.8 [3.2-4.4]	6.6 [6.1-7.2]	72.8
South Atlantic	3.8 [3.4-4.1]	6.4 [6.1-6.7]	68.5
East South Central	4.4 [3.8-5.1]	8.1 [7.5-8.8]	82.9
West South Central	1.8 [1.5-2.1]	4.5 [4.2-4.8]	148.1
Mountain	4.3 [3.7-4.9]	6.5 [6.0-7.0]	51.2
Pacific	2.4 [2.1-2.7]	5.6 [5.3-5.9]	130.0

**Figure 4:**
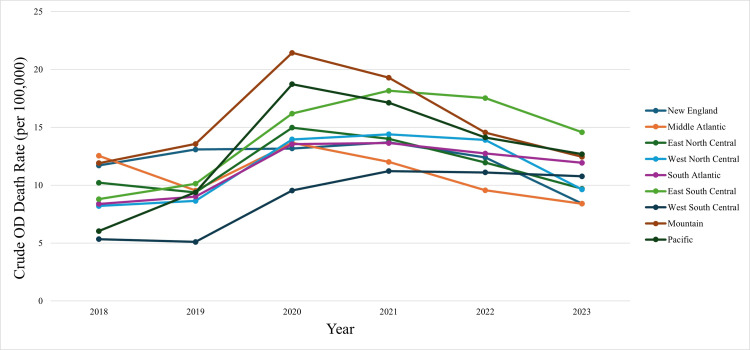
Trends in crude overdose (OD) death rates among male youth and young adults aged 10 to 24 from 2018 to 2023 by census division. Crude death rates are based on population counts of >1,000,000 individuals and presented per 100,000 individuals.

**Figure 5:**
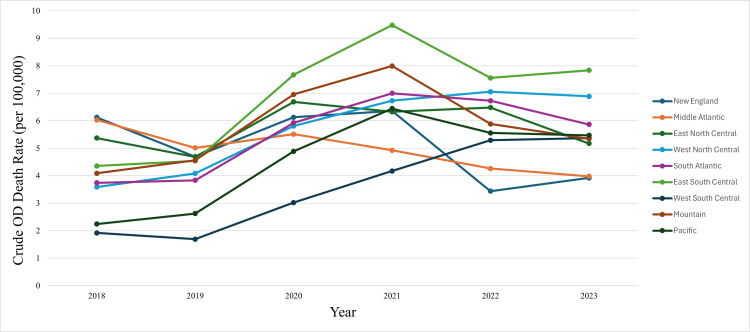
Trends in crude overdose (OD) death rates among female youth and young adults aged 10 to 24 from 2018 to 2023 by census division. Crude death rates are based on population counts of >1,000,000 individuals and presented per 100,000 individuals.

Temporal analyses further underscore these patterns. From the pre-pandemic years 2018-2019, crude YYA OD death rates were relatively stable across most regions of the U.S. Both male and female YYAs across US census divisions experienced increases in OD death rates in 2020 and 2021, with decreases in OD death rates for several divisions since the peak of the COVID-19 pandemic (Figures 4, 5). The highest rate recorded among males was in the Mountain region in 2020. As of 2023, the highest OD death rates among YYA males were in the East South Central region (Figure 4). Moreover, the East South Central region had the highest peak of crude OD death rate among YYA females in 2021, and this region continued to have the highest OD death rates among YYA females in 2023 (Figure 5). 

Sex-stratified analyses revealed that while males consistently demonstrated overall higher crude OD death rates than females throughout the study period, the relative increase was greater for females in regions experiencing an increase in OD death rates, except for the East North Central region (Tables 2, 3). This narrowing sex gap suggests that female YYAs, who historically have had lower OD mortality, are increasingly vulnerable in the current peri/post-pandemic landscape (Figures 3, 5).

**Figure 6:**
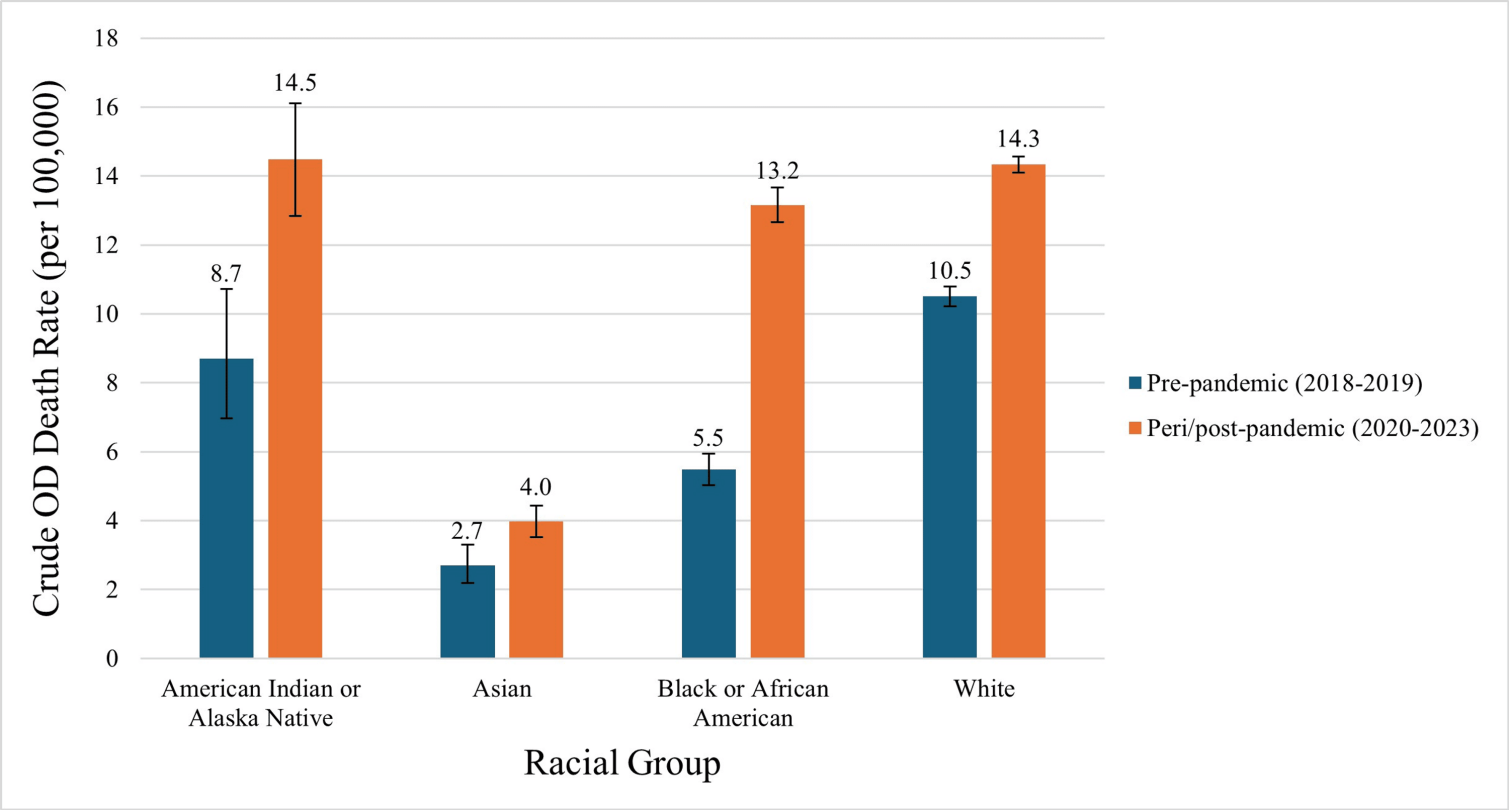
Pre-pandemic (2018-2019) and peri/post-pandemic (2020-2023) overdose (OD) death rates among youth and young adult males aged 10 to 24 years by racial grouping. Crude death rates are based on population counts of >1,000,000 individuals and presented per 100,000 individuals with 95% confidence intervals.

**Figure 7:**
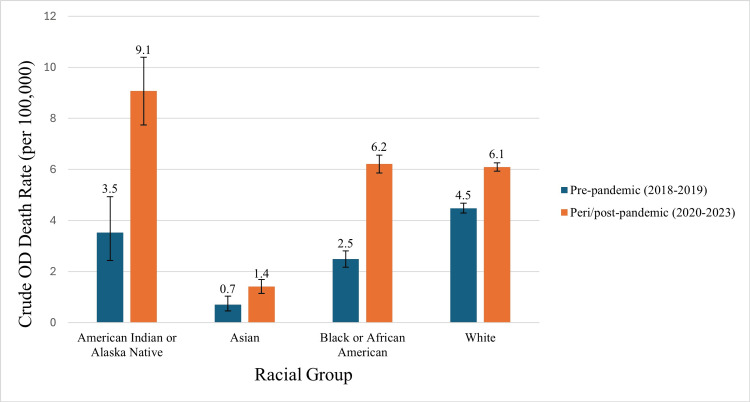
Pre-pandemic (2018-2019) and peri/post-pandemic (2020-2023) overdose (OD) death rates among youth and young adult females aged 10 to 24 years by racial grouping. Crude death rates are based on population counts of >950,000 individuals and presented per 100,000 individuals with 95% confidence intervals.

**Figure 8:**
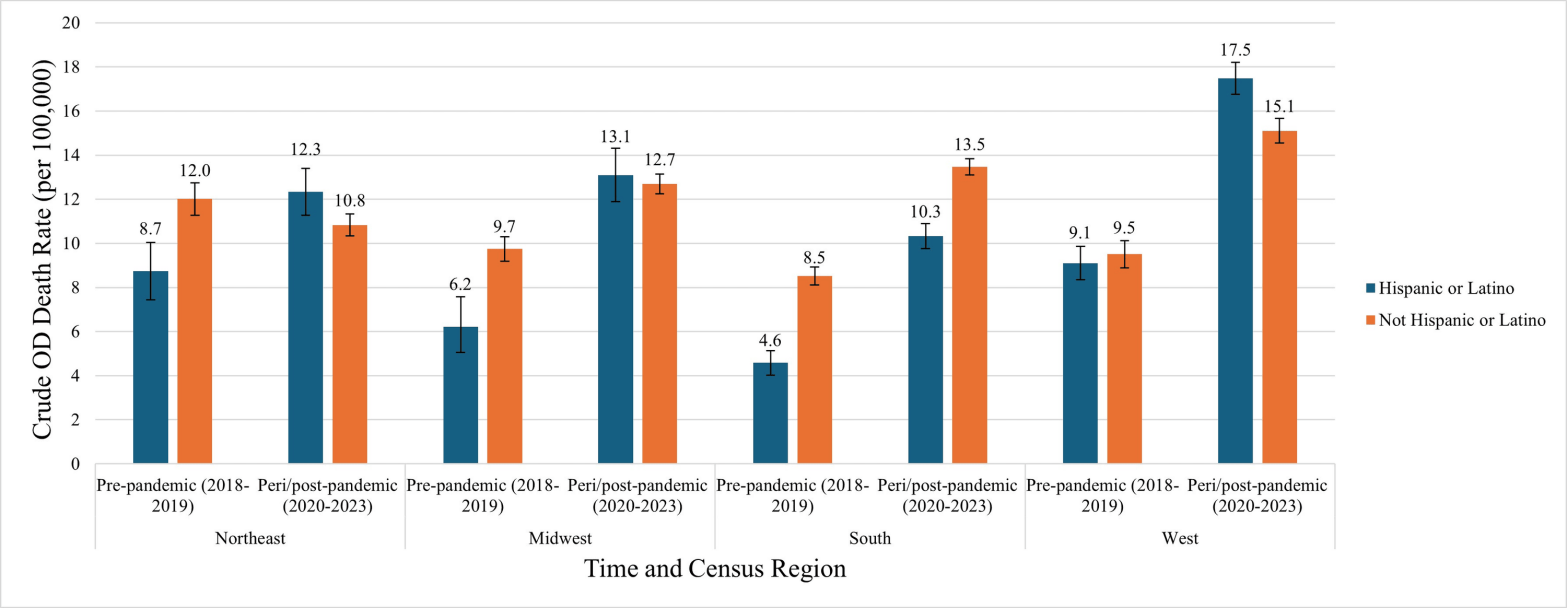
Pre-pandemic (2018-2019) and peri/post-pandemic (2020-2023) overdose (OD) death rates among male youth and young adults aged 10 to 24 years by US census region and ethnicity. Crude death rates are based on population counts of >1,000,000 individuals and presented per 100,000 individuals with 95% confidence intervals.

**Figure 9:**
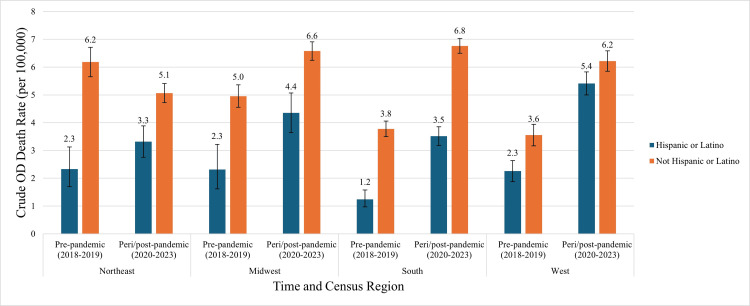
Pre-pandemic (2018-2019) and peri/post-pandemic (2020-2023) overdose (OD) death rates among female youth and young adults aged 10 to 24 years by US census region and ethnicity. Crude death rates are based on population counts of >1,000,000 individuals and presented per 100,000 individuals with 95% confidence intervals.

Racial and ethnic subgroup analyses further highlighted disparities. While all races experienced increased OD death rates from pre-pandemic to peri/post-pandemic years, AI/AN YYAs experienced the highest overall crude death rates nationally across both sexes in the peri/post-pandemic period (Figures 6, 7). AI/AN males experienced an increase in OD death rate from 8.7 [7.0-10.7] pre-pandemic to 14.5 [12.8-16.1] peri/post-pandemic, while female OD death rates increased from 3.5 [2.4-4.9] to 9.1 [7.7-10.4] from pre- to peri/post-pandemic. Black or African American YYAs exhibited an over twofold increase in crude OD mortality over the study period, from 5.5 [5.0-5.9] to 13.2 [12.6-13.7] in males and 2.5 [2.2-2.8] to 6.2 [5.9-6.6] per 100,000 in females, reflecting a rapidly worsening burden in this population. Asian YYAs also experienced nearly a doubling in crude death rates from pre-pandemic to peri/post-pandemic years, with male rates increasing from 2.7 [2.2-3.3] to 4.0 [3.5-4.4] and female rates increasing from 0.7 [0.5-1.0] to 1.4 [1.1-1.7] (Figures 6, 7). Hispanic YYAs also experienced increasing OD mortality rates across the pandemic. For females, peri/post-pandemic OD death rates among Hispanics remained lower than those of their non-Hispanic or Latino counterparts in all census regions. However, Hispanic or Latino males experienced higher OD death rates in the peri/post-pandemic period in the Northeast, Midwest, and West regions (Figures 8, 9).

## Discussion

The OD epidemic has evolved in recent US history, beginning with prescription opioid-related misuse and evolving into synthetic opioids, such as fentanyl. Most recently, the epidemic has entered the “fourth wave,” characterized by fentanyl increasingly being contaminated with veterinary sedatives such as xylazine and medetomidine [7]. Given the influx of polysubstance and evolving OD risks as a result of adulteration, understanding national drug-related deaths, particularly among younger individuals, is important to inform future research, clinical practice, and policy decisions.

Using the CDC WONDER data, this study aimed to offer insight into the national trends of unintentional OD deaths among YYAs, representing the population of 10- to 24-year-olds. The data support the hypothesis that YYA unintentional OD deaths peaked during the COVID-19 pandemic, and for the majority of the country, these rates continue to be higher post-pandemic than they were pre-pandemic. The East South Central region, comprised of Alabama, Kentucky, Mississippi, and Tennessee, continues to have the highest rates of YYA OD deaths in the country. AI/AN and Black or African American YYAs experienced the largest increase in rates of unintentional OD from 2018 to 2023. Finally, while YYA males overall had higher rates of fatal OD, the percent changes of crude OD death rates were generally higher among YYA females.

Trends in regional and geographical OD rates continue to be debated throughout the literature. Many studies review OD death rates, among all ages, by state or limited regionality. For example, Lyle Cooper et al. found that in 2016, the highest rates of OD-related deaths occurred in West Virginia, New Hampshire, Ohio, Maryland, and Massachusetts, with West Virginia having the highest rates of semisynthetic opioid OD [6]. Zhao et al. found that the states with the highest age-standardized mortality rates during the pandemic included West Virginia, the District of Columbia, Louisiana, and Tennessee [2]. Specifically, among the pediatric population under 12 years old, Kelly et al. found the highest rates of pediatric ODs in the Appalachia region, and states including Oklahoma and Montana [11]. The Great Plains region, and the individual states New York, New Jersey, Virginia, Texas, and California, had the lowest pediatric OD rates [10]. Regional differences can also be seen among specific groups of people. Tipps et al. found significant regional differences among AI/AN populations [22].

While regional effects may vary based on study method parameters, cohort age, and time, there is evidence that areas spending more on public welfare and Medicaid have significantly lower OD mortality rates, especially among pediatric and adolescent populations [11]. Areas with Good Samaritan Laws also had significantly reduced county-level pediatric OD rates [11]. Public resources focused on YYAs are necessary for reducing ODs. In regions with the highest observed rates, such as the East South Central division, these strategies may be complemented by expanding naloxone training and access to treatment, especially since it is estimated that only about 3% of those aged 10-19 years who die from fatal ODs were actively engaged in substance use disorder treatment [23]. Therefore, investigating medical, legal, and sociopolitical paradigms through collective collaborative efforts among experts across professions will be beneficial in targeting areas most in need.

Males tend to experience higher rates of OD than females [2,23-25]. Prior to the pandemic, from 1999 to 2016, Gaither et al. found that roughly 73% of fatal opioid ODs in children and adolescents were males [25]. An explanation for this difference may be due to behavioral differences, as male adolescents tend to engage in more risk-taking behaviors [26]. Freibott et al. also found that females had a greater willingness to intervene during an OD compared to males [27]. Nevertheless, while males overall have higher rates of OD, this study found that YYA females are experiencing a higher percent change in OD rate compared to males, especially throughout the pandemic. This finding is supported by Zhao et al., who also reported a higher excess mortality rate among females compared to males during the pandemic [2]. A potential explanation for this could be the telescoping effect, in which females have been described to have accelerated progression of misuse and progress faster from first drug exposure to entering treatment for substance use in the future [28]. While it is unclear whether the telescoping effect is driving the observed trends, the higher percent change of OD death rates among females is a concerning finding that warrants further investigation.

Rates of drug overdose deaths have been rising more steeply among minority racial and ethnic populations, especially within the AI/AN and Black and African American communities. Friedman and Hansen found that since 2015, the OD death rates among Black communities have risen rapidly, and in 2019, the rate of OD among Black individuals surpassed that of White individuals [29]. Such effects have continued to persist, particularly in the current “fourth wave” of the opioid crisis. In a 2024 study by Friedman et al., the drug OD death rate among Black Americans had reached 1.4 times the rate among White Americans by 2022, and the rate for AI/AN populations had reached 1.8 times higher [30]. Given these racial disparities, targeted intervention that includes expanded access to substance use disorder treatment facilities as well as further public education in drug use dangers could become viable changes to reduce unintentional OD deaths, particularly among the vulnerable YYA population.

This study has several limitations. While CDC WONDER offers valuable national public health data, the accuracy of its mortality data depends on death reporting, which may vary across states, regions, and ethnicities, introducing potential reporting and misclassification bias. In addition to reporting biases, data suppression may disproportionately affect smaller populations. This is particularly relevant for NH/OPI and AI/AN groups, as well as rural regions, where population counts may be low, but the relative impact of unintentional OD could be high. Given this study’s use of population-level data, it is also subject to ecological fallacy, and observed trends should not be interpreted as individual-level risk factors. To adhere to CDC WONDER use guidelines and provide a robust study population across US census divisions and various racial/ethnic groups, this study aggregated those aged 10-24 years, which may mask developmental differences between children, adolescents, and young adults. Specifically, deaths occurring in those aged 10-14 may reflect accidental ingestion, whereas deaths occurring in those aged 20-24 could be due to deliberate substance use. Future studies should aim to perform sensitivity analyses across 5-year age strata and evaluate whether these trends differ across developmental subgroups.

This study was also designed to be a purely descriptive ecological cross-sectional time-trend analysis, with no inferential statistics or adjustment for potential confounders such as differential healthcare access and state-level policies, which is a limitation. Furthermore, given that the data returned by CDC WONDER did not include individual-level toxicology reports and more granular ICD-10 (International Classification of Diseases, 10th Revision) codes that distinguish deaths by drug type, it was not possible to confirm the exact extent to which the observed OD trends were driven by fentanyl, heroin, prescription opioids, other drugs, or polysubstance use. Lastly, the definition of years 2018-2019 as “pre-pandemic” and 2020-2023 as “peri/post-pandemic” likely oversimplifies the temporal nuances of the COVID-19 pandemic and obscures monthly fluctuations in OD trends during that time. It must also be noted, however, that changes in OD deaths observed in this study should not be interpreted as being directly attributable to the COVID-19 pandemic, given the retrospective, non-inferential nature. Based on these limitations, this study’s findings should be interpreted as national population-level surveillance data that identify trends and disparities in unintentional OD deaths, rather than establish causality.

Despite these limitations, this study provides a foundational report on who is most affected within the country’s YYA population and the areas most affected, emphasizing the need for targeted intervention. Future studies should aim to inform prevention and treatment by elucidating why these trends persist, what optimal policy changes can be enacted, and how to enhance access to treatment despite disparities.

## Conclusions

Youth and young adults aged 10-24 years old continue to be impacted by the ongoing drug OD crisis, signifying a public health concern that warrants ongoing attention. Rates of unintentional OD deaths remain elevated above pre-pandemic levels despite decreasing since the peak of the pandemic. These OD trends varied by region, sex, race, and ethnicity. Developing group- and region-specific prevention and treatment strategies, particularly for AI/AN and Black and African American YYAs, will allow for maximization of resource allocation, in hopes of reducing preventable, unintentional deaths among this vulnerable population. Clinicians and healthcare workers, who are often on the frontline of managing patients suffering from drug OD, should also be aware of these trends when developing prevention and treatment strategies for YYAs.
